# Meningiomas classified as grade 2 due to brain invasion alone have better progression-free survival than other atypical meningiomas

**DOI:** 10.1007/s11060-026-05783-1

**Published:** 2026-09-11

**Authors:** Salem M. Tos, Sofia Kollia, Georgios Mantziaris, Georgios A. Maragkos, Carson Brantley, Jared H. Chung, Maria-Beatriz Lopes, Ashok R. Asthagiri

**Affiliations:** 1 Department of Neurological Surgery, University of Virginia, Charlottesville, VA USA; 2 School of Medicine and Health Sciences, The George Washington University, Washington, D.C USA; 3 Department of Pathology, University of Virginia, Charlottesville, VA USA

**Keywords:** Brain invasion, Atypical meningioma, WHO Grade 2, Brain invasion otherwise benign (BIOB), Tumor progression, Time to progression

## Abstract

**Background:**

Brain invasion (BI) was incorporated as a major criterion for grade 2 meningiomas in the 2016 WHO guidelines. However, its prognostic value remains controversial due to variability in surgical sampling and histopathologic interpretation, as well as BI may be the consequence of mechanical forces related more with the size of the tumor than the biologic presence of an innately more aggressive biologic lesion. Despite advances in molecular characterization of meningiomas, the relevance of BI as an independent prognostic indicator remains unresolved. We sought to determine whether BI alone, taken out of context of other parameters, particularly tumor size, truly represents a more aggressive standalone feature, by comparing time-to-progression (TTP) in atypical meningiomas classified as such due to BI alone versus other atypical meningiomas.

**Methods:**

A single-institutional retrospective cohort study of patients with resected meningiomas from 2003 to 2023 was performed. Pathology reports were reviewed and tumors were re-graded using current WHO criteria. Only WHO Grade 2 patients were included in the study. Patients were then divided into two cohorts: The BIOB (Brain Invasion Otherwise Benign) group and “other criteria” (OC) group. Primary outcome was TTR and association of factors with TTR were assessed using Cox regression.

**Results:**

131 patients with a mean age of 59.2 ± 15.9 years were included. Median (IQR) preoperative tumor volume was 34.3 cm^3^ (16-57.9). Gross total resection was achieved in 69 (53%) tumors. 18 patients (14%) had adjuvant radiation. BI was noted in 78 (60%) patients, 45 (34%) of which were classified as BIOB. Mean radiographic follow-up was 74 ± 56 months. Progression was noted in 40 (31%) patients at a mean of 40 ± 39 months post-resection. Progression occurred in 17.8% of BIOB patients (8/45) and 37.2% of OC patients (32/86). Baseline demographic, radiologic, and treatment characteristics did not differ significantly between BIOB and OC groups (all *P* > 0.3). On univariate Cox regression, TTR was associated with the BIOB group (Hazard Ratio [HR] = 0.44, *P* = 0.036), GTR (HR = 0.29, *P* < 0.001), and residual volume (HR = 1.04, *P* = 0.002). On multivariable Cox regression, BIOB was associated with longer TTR (HR = 0.44, *P* = 0.041), when controlling for GTR and posterior fossa location.

**Conclusions:**

Atypical meningiomas secondary to brain invasion alone were associated with significantly longer TTR than tumors with other Grade 2 criteria. This was especially robust for larger BI-only tumors. Further research is needed to delineate if brain invasion should truly be a standalone criterion for Grade 2 classification.

**Supplementary Information:**

The online version contains supplementary material available at https://doi.org/10.1007/s11060-026-05783-1.

## Introduction

Meningiomas are the most common primary intracranial tumors, and their World Health Organization (WHO) grade, benign (grade 1) or atypical (grade 2), directly informs postoperative management [1]. Atypical meningiomas are defined by increased mitotic activity, a combination of specific histomorphological features, or brain invasion (BI) into adjacent brain parenchyma [2]. More recently, the cIMPACT-NOW consortium has highlighted the growing role of molecular markers, including CDKN2A/CDKN2B homozygous deletion, TERT promoter alterations, and specific chromosomal arm losses (e.g., 1p, 22q), in refining risk stratification for meningiomas, including BI-defined cases; despite this progress, considerable controversy persists regarding the independent prognostic value of BI as a grading criterion [3]. The 2016 WHO guidelines edition and subsequent 2021 update for CNS tumors integrated parenchymal invasion as a stand-alone criterion for the assignment of atypical, WHO grade 2 meningioma [4, 5]. This change in meningioma grading was derived from a single-center, retrospective study that evaluated 581 patients with histopathologic confirmation of meningioma; of the 76 cases where brain invasion (BI) could be assessed in the tumor specimen, 22 cases were classified as brain invasive and were compared to 54 cases with the absence of brain invasion [6]. The results of this study indicated that brain invasion was strongly correlated with lower progression-free survival rates [6].

However, neither surgical sampling nor neuropathological analyses are standardized; differences in neurosurgical sampling can lead to significant loss of tumor tissue prior to neuropathological analyses. Following these changes in WHO grading, a number of studies have contested the inclusion of brain invasion as a standalone criterion [7–10]. This retrospective study assesses the impact of brain invasion on meningioma progression for WHO grade 2 meningiomas and analyzes factors that may help to determine its importance at a US academic center.

## Methods

### Study design, population and outcomes

The current study is a single-center retrospective cohort study. Patients with surgical resection of WHO Grade 2 meningiomas were reviewed from a 20-year (2003–2023) period. To account for the change in classification, and to ensure all tumors were graded on the same criteria, pathology slides were reviewed and upgraded or downgraded based on the 2016 WHO classification criteria [4]. The 2016 classification incorporated BI as an independent criterion for a tumor to be considered WHO Grade 2. After recategorization according to the 2016 WHO classification criteria, patients were placed into two study groups. Patients whose meningioma had been re-graded to WHO Grade 2 solely because of the presence of BI were considered part of the BIOB group. All other tumors which met at least one other criterion for a WHO Grade 2 meningioma (for example, mitotic index ≥ 4 mitoses/10 HPFs, minor atypical features such as hypercellularity, macronucleoli, small cell change and patternless/ sheet-like growth, and foci of spontaneous, Predominant chordoid or clear cell morphology, or combination of these with BI) were considered part of the Other Criteria Group. Patients with pathology-proven WHO Grade 2 meningiomas who underwent surgical resection with follow up and adequate peri-operative imaging data were included. For purposes of this study, the pathological review included re-graded pathology slides to reclassify tumors into the study groups. BI was defined histologically as the presence of tumor cells infiltrating into the adjacent brain parenchyma without an intervening connective tissue layer, as evaluated on hematoxylin-eosin and van Gieson-Elastica–stained slides [10]. Patients were excluded if they had received any pre-operative or other prior treatment for the tumor (for example, radiation therapy), had a spinal meningioma, or if follow up duration was < 3 months. The STROBE (Strengthening the Reporting of Observational Studies in Epidemiology) checklist was used to ensure clarity, transparency, and rigor in study design and reporting [11].

**Fig. 1:**
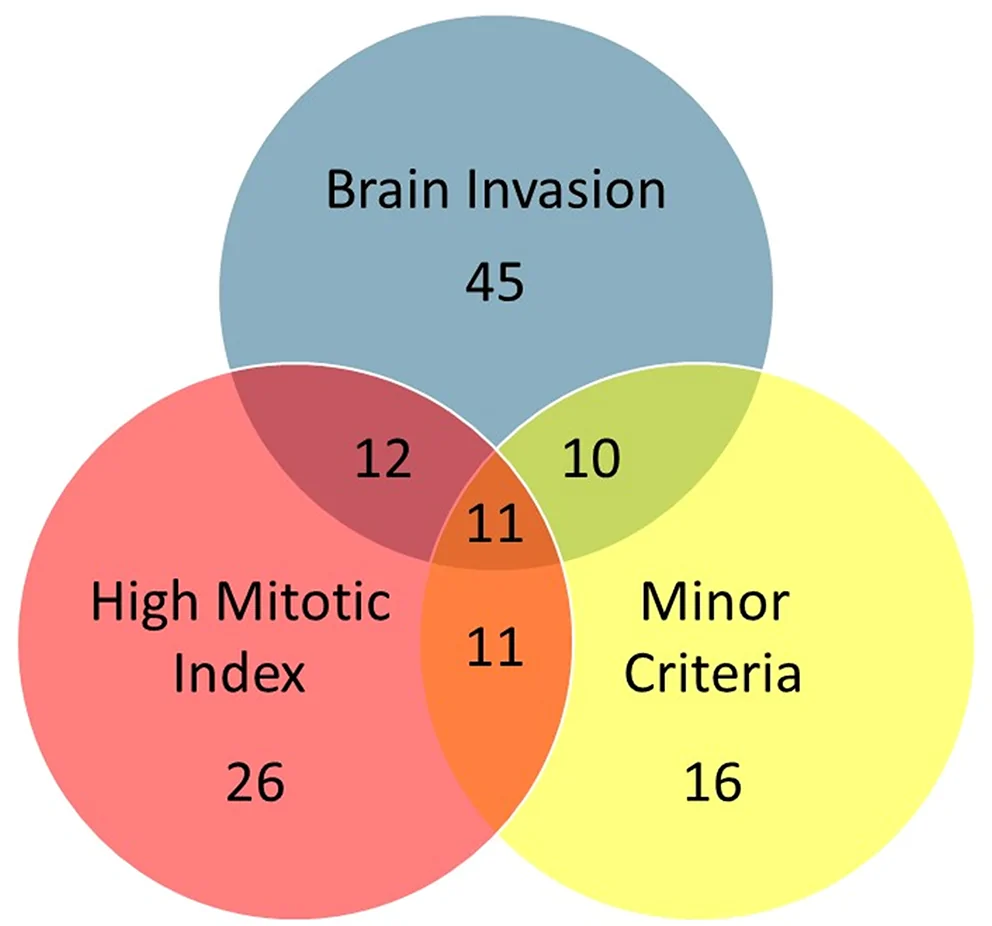
Patient-level distribution of WHO grade 2 meningioma classification criteria. The Venn diagram (left) illustrates the overlap between patients meeting histopathologic criteria: Brain Invasion (red), Mitotic Index ≥ 4 (blue), and Minor Criteria (yellow); numbers represent patients fulfilling individual or overlapping criteria. The pie chart (right) shows the proportion of patients with Brain Invasion as the sole criterion (*n* = 45, 34%) versus those meeting any other criteria combinations (*n* = 86, 66%). Total cohort: 131 patients

Of the 131 patients with WHO Grade 2 meningiomas, 78 tumors demonstrated brain invasion. We further stratified these cases into two groups: 45 tumors met Grade 2 criteria based solely on brain invasion (“BI-only” group), while the remaining 86 met criteria based on other features (high mitotic index and/or minor atypical criteria), with or without brain invasion (“Other Criteria” group). The distribution and overlap of these histopathologic features are illustrated in the Venn diagram (Fig. 1).

### Data variables and outcomes

Patient age, sex, and tumor location were collected. Tumor location was further stratified to include convexity, skull base, or posterior fossa. Tumor characteristics were collected including preoperative tumor volume. Preoperative imaging was also reviewed for presence of surrounding edema. Surgical details were also collected, including the extent of resection which was dichotomized as either gross total resection (GTR) or subtotal resection. Volume of residual tumor after surgery was also collected. Use of adjuvant radiation therapy post-operatively was also collected. The primary outcome was time-to-progression (TTP), defined as the interval from the date of surgical resection to the date of radiographic evidence of tumor progression on follow-up imaging. Secondary outcomes included progression rate and correlation of progression to clinical/pathological/treatment-related factors. These outcomes were selected to gain a deeper understanding of the clinical course of BIOB meningiomas as compared to tumors otherwise meeting criteria for WHO Grade 2 classification.

### Statistical analysis

Statistical analyses were conducted using R Studio. Descriptive statistics were used to summarize baseline characteristics, including demographics, tumor features, and treatment modalities. Continuous variables were summarized using means with standard deviations or medians with interquartile ranges, depending on distribution, and categorical variables were summarized as frequency and percentages. Univariate analyses were performed to identify potential associations between individual variables and tumor progression. Categorical variables were compared using chi-square or Fisher’s exact tests as appropriate, and continuous variables were compared using t-tests or Mann-Whitney U tests, depending on their distribution. Cox proportional hazards regression was used to evaluate the association between individual variables and time-to-progression (TTP). Variables with a significant association with tumor progression in the univariate analyses were entered into multivariable Cox regression to adjust for confounding variables and identify independent predictors of progression. Results were presented as hazard ratios (HRs) with 95% confidence intervals (CIs) and p-values. Sensitivity analyses were performed to assess the robustness of the findings, including reclassification of small tumors based on their actual grades and excluding patients whose tumor met overlapping criteria or received adjuvant radiation therapy and evaluation of adjuvant radiation therapy under multiple model specifications. A pre-specified multivariable Cox model was also constructed incorporating age, tumor location, edema, extent of resection, and adjuvant radiation therapy. Kaplan-Meier survival analysis was used to compare progression free survival between tumor classification groups. Variables with missing data were noted but overall data completeness was high.

## Results

### Patient demographics and tumor characteristics

A total of 131 patients with WHO Grade 2 meningiomas were included in the study. (Table 1) The mean age of the cohort was 59.2 ± 15.9 years, and the majority of patients were female (60%). The most common tumor location was convexity (74%), followed by skull base (22%) and posterior fossa (4%). The median preoperative tumor volume was 34.3 cc (IQR: 16–57.9). Preoperative surrounding edema was present in 92% of cases and irregular tumor borders were noted in 96%. Bone invasion was identified in 8% of cases.

**Table 1:** Summary statistics

Variable	Patients (%), *N* = 131	Missing Data
*Demographics*		
Age, years (mean ± SD)	59.2 ± 15.9	0
Sex		0
Male	52 (40)	
Female	79 (60)	
*Preoperative Tumor Characteristics*		
Preoperative diameter, mm (mean ± SD)	49.8 ± 16.5	1
Preoperative volume, cc [median, IQR]	34.3 [16, 57.9]	2
Side		0
Midline	17 (13)	
Left	69 (53)	
Right	45 (34)	
Location		0
Convexity	97 (74)	
Skull base	29 (22)	
Posterior fossa	5 (4)	
Presence of surrounding edema	121 (92)	0
Irregular tumor borders	125 (96)	1
Irregular enhancement	127 (98)	1
Bone invasion	10 (8)	1
*Treatment Data*		
Simpson grade		4
1	4 (3)	
2	61 (48)	
3	6 (5)	
4	54 (43)	
5	2 (2)	
Gross total resection (Simpson Grade 1–2)	69 (53)	0
Postoperative residual diameter, mm [median, IQR]	0 [0, 22.8]	1
Postoperative residual volume, cc [median, IQR]	0 [0, 2.7]	1
Adjuvant radiation	18 (14)	0
Time to adjuvant radiation, months (mean ± SD)	2.9 ± 2	1
*Pathology Data*		
Mitotic count ≥ 4–19 mitoses/10 hpf	60 (46)	0
Choroid-type meningioma	8 (6)	0
Clear cell-type meningioma	8 (6)	0
Grade 2 minor criteria	38 (29)	0
Brain invasion	78 (60)	0
Deemed Grade 2 due to “brain invasion” alone	45 (34)	0
Deemed Grade 2 due to “brain invasion” alone, and preoperative volume larger than 25th percentile (16 cc)	34 (26)	2
*Follow-up*		
Radiographic follow-up, months (mean ± SD)	74.3 ± 55.7	0
Clinical follow-up, months (mean ± SD)	74.9 ± 58.5	0
Recurrence	40 (31)	0
Time to recurrence, months (mean ± SD)	40.2 ± 38.7	0
Tumor volume at recurrence, cc (mean ± SD)	17.4 ± 20.1	16
Retreatment type		0
SRS	24 (18)	
Resection	8 (6)	
FSRT	4 (3)	
IMRT	2 (2)	
None	1 (1)	
Time to retreatment, months (mean ± SD)	47.2 ± 39.2	2
Multiple recurrences	17 (43)	0
Number of recurrences to date [median, IQR]	2 [2, 4]	0
Mortality during follow-up period	20 (15)	0

BI was noted in 60% of tumors, and 34% of tumors met WHO Grade 2 criteria based on the presence of brain invasion only. Among these, 26% had a preoperative tumor volume > the 25th percentile (≥ 16 cc). Minor histopathological criteria for Grade 2 meningioma (hypercellularity, macronucleoli, and sheet-like growth) were noted in 29% of cases, while mitotic count ≥ 4/10 high power fields was present in 46%. Chordoid and clear-cell meningiomas, which both may present with BI, were 6% of the entire cohort, each.

Baseline characteristics between diagnostic groups.

Baseline demographic, radiologic, and treatment characteristics were compared between the BIOB and OC groups (Table 2). None of the variables examined, including age, sex, tumor location, preoperative tumor volume, edema, gross total resection rate, residual tumor volume, adjuvant radiation therapy use, or radiographic follow-up duration, differed significantly between groups (all *p* > 0.3), other than a trend toward younger age in the BIOB group (55.7 vs. 61.1 years, *p* = 0.055).

**Table 2:** Baseline characteristics, BIOB vs. OC

Characteristic	BIOB (*n* = 45)	OC (*n* = 86)	*P*-value
Age, years, mean ± SD	55.7 ± 14.4	61.1 ± 16.5	0.055
Female sex, n (%)	26 (58%)	53 (62%)	0.811
Location: Convexity / Skull base / Post. fossa, n (%)	31 (69) / 11 (24) / 3 (7)	66 (77) / 18 (21) / 2 (2)	0.393
Preop tumor volume, cc, mean ± SD	43.2 ± 34.4	40.9 ± 32.5	0.717
Preop tumor diameter, mm, mean ± SD	49.0 ± 15.0	50.2 ± 17.3	0.691
Preop edema present, n (%)	43 (96%)	78 (91%)	0.493
Gross total resection, n (%)	26 (58%)	42 (49%)	0.430
Residual tumor volume, cc, mean ± SD	1.8 ± 4.0	3.1 ± 8.5	0.255
Adjuvant radiation therapy, n (%)	5 (11%)	13 (15%)	0.715
Radiographic follow-up, months, mean ± SD	68.3 ± 51.1	77.4 ± 58.0	0.362

None of the surgical or anatomic variables differ significantly between groups (all *p* > 0.3); BIOB patients are numerically younger (*p* = 0.055).

### Treatment characteristics

GTR was performed in 53% of cases and 47% of cases were subtotal resections. The median postoperative residual tumor volume was 0 cc (IQR: 0–2.7). 14% of patients received adjuvant radiation therapy with a mean time to treatment of 2.9 ± 2 months.

### Outcomes

The mean length of radiographic follow-up was 74.3 ± 55.7 months, and the mean length of clinical follow-up was 74.9 ± 58.5 months. Tumor progression was observed in 31% of the entire cohort, with a mean time to progression of 40.2 ± 38.7 months. The mean tumor volume at the time of progression was 17.4 ± 20.1 cc. Retreatment modalities included SRS (18%), repeat surgical resection (6%), FSRT (3%), and IMRT (2%). Mortality was observed in 15% of the cohort during the follow-up period.

### Factors associated with progression vs. non-progression

**Fig. 2:**
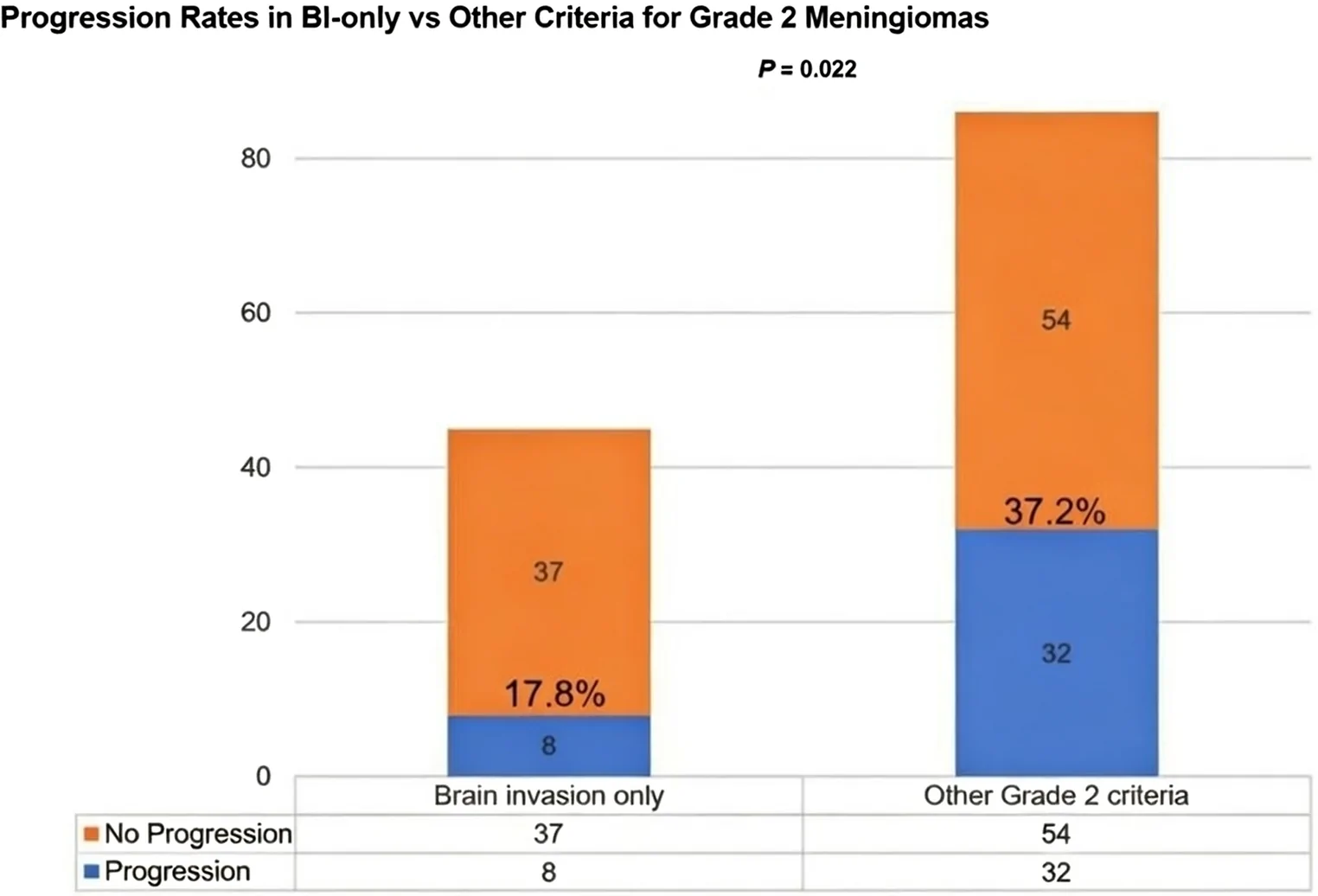
Bar chart comparing recurrence rates between BIOB and other Grade 2 criteria (OC) Grade 2 meningiomas. Recurrence was significantly lower in the BIOB group (17.8%, 8/45) compared to the OC group (37.2%, 32/86) (*p* = 0.022)

**Fig. 3:**
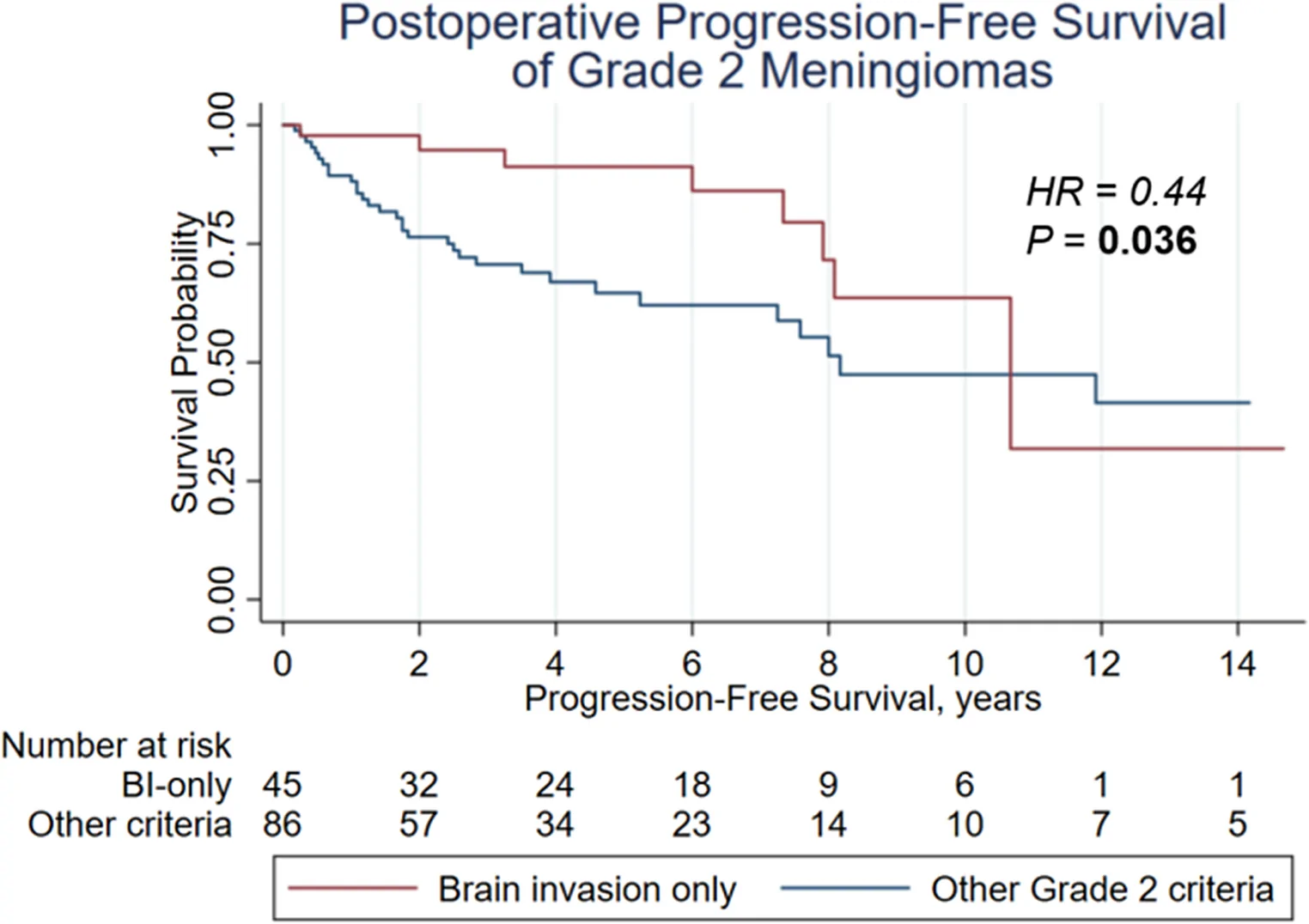
Kaplan–Meier curve showing recurrence -free survival in WHO Grade 2 meningiomas. Patients with tumors classified as Grade 2 due to brain invasion only (BIOB) demonstrated significantly longer recurrence-free survival compared to those meeting other Grade 2 criteria (OC) (*p* = 0.036)

Dividing the cohort into those that had progression vs. those that did not, we identified multiple factors significantly associated with tumor progression (Table 3). Patients who had a GTR were significantly less likely to have tumor recurrence (25% vs. 64%, *p* < 0.001). Similarly, postoperative residual tumor volume was significant predictor of progression (*p* = 0.025). Histopathological factors were also significant. Tumors meeting the Grade 2 criteria due to minor features had a significantly higher rate of progression (53%, *p* < 0.001). On the other hand, tumors that were classified as Grade 2 due to BI alone had a significantly lower progression rate (17.8% vs. 37.2%, *p* = 0.022; Fig. 2). This remained significant when limiting analysis to patients with a preoperative tumor volume > the 25th percentile (16 cc, *p* = 0.05). To further examine whether tumor size influenced progression patterns, we stratified tumors into small (< 16 cc, corresponding to the 25th percentile of preoperative volume) and large (≥ 16 cc) categories and compared progression rates between diagnostic groups. Among small tumors, progression rates were similar between meningiomas deemed Grade 2 due to BI alone and those upgraded due to at least one other criterion (18.2% vs. 33.3%; Fisher’s exact *p* = 0.441). The subgroup of small tumors was numerically limited, which may have contributed to the absence of a detectable statistical difference. In contrast, among larger tumors (≥ 16 cc), the BI alone group demonstrated markedly lower progression rates compared with tumors meeting at least one other Grade 2 criterion (17.6% vs. 39.7%; χ² *p* = 0.046). (Table 4). In univariate Cox regression based on time to progression (Table 5), GTR was strongly associated with a reduced hazard of recurrence (HR: 0.29, 95% CI: 0.14–0.59, *p* < 0.001). Postoperative residual tumor volume (HR: 1.04, 95% CI: 1.01–1.07, *p* = 0.002) was associated with an increased hazard of progression. Grade 2 due to BI alone had a significantly lower risk of progression (HR: 0.44, 95% CI: 0.2–0.95, *p* = 0.036). This difference is also reflected in the Kaplan–Meier survival analysis, where the BI-only group demonstrated significantly longer progression-free survival (Fig. 3). Minor Grade 2 criteria was associated with higher progression risk (HR: 3.31, 95% CI: 1.78–6.19, *p* < 0.001).

**Table 3:** Univariate analysis based on incidence of recurrence

Variable	No recurrence (%), *n* = 91 (69%)	Recurrence (%), *n* = 40 (31%)	*P*-value	Missing Data
*Demographics*				
Age, years (mean ± SD)	59 ± 17	60 ± 13	0.56	0
Sex			0.663	0
Male	35 (38)	17 (43)		
Female	56 (62)	23 (58)		
*Preoperative Tumor Characteristics*				
Preoperative volume, cc [median, IQR]	33.1 [15.8, 54.1]	35.3 [16.9, 62]	0.57	2
Side			0.18	0
Midline	9 (10)	8 (20)		
Left	52 (57)	17 (43)		
Right	30 (33)	15 (38)		
Location			0.33	0
Convexity	70 (77)	27 (68)		
Skull base	17 (19)	12 (30)		
Posterior fossa	4 (4)	1 (3)		
Presence of surrounding edema	84 (92)	37 (93)	0.97	0
Irregular tumor borders	86 (96)	39 (98)	0.6	1
Irregular enhancement	88 (98)	39 (98)	0.92	1
Bone invasion	7 (8)	3 (8)	0.96	1
*Treatment Data*				
Gross total resection (Simpson Grade 1–2)	58 (64)	10 (25)	**< 0.001**	0
Postoperative residual volume, cc [median, IQR]	0 [0, 1.2]	1.4 [0, 6]	**0.025**	1
Adjuvant radiation	14 (15)	4 (10)	0.41	0
Time to adjuvant radiation, months (mean ± SD)	3 ± 2	3 ± 3	0.69	1
*Pathology Data*				
Mitotic count ≥ 4–19 mitoses/10 hpf	38 (42)	22 (55)	0.16	0
Choroid-type meningioma	4 (4)	4 (10)	0.22	0
Clear cell-type meningioma	8 (9)	0 (0)	0.053	0
Grade 2 minor criteria	17 (19)	21 (53)	0	0
Brain invasion	58 (64)	20 (50)	0.14	0
Deemed Grade 2 due to “brain invasion” alone	37 (41)	8 (20)	**0.022**	0
Deemed Grade 2 due to “brain invasion” alone, and preoperative volume larger than 25th percentile (16 cc)	28 (31)	6 (15)	**0.05**	2
*Follow-up Data*				
Mortality during follow-up period	11 (12)	9 (23)	0.13	0

**Table 4:** Recurrence rate by tumor size and grading group

Tumor Size Category	Group	No Recurrence *n* (%)	Recurrence *n* (%)	*p*-value
Small tumors (< 16 cc)	Deemed Grade 2 due to “brain invasion” alone	9 (81.8%)	2 (18.2%)	0.4414
	Deemed Grade 2 due to at least one other criterion	14 (66.7%)	7 (33.3%)	
Large tumors (≥ 16 cc)	Deemed Grade 2 due to “brain invasion” alone	28 (82.4%)	6 (17.6%)	**0.0463**
	Deemed Grade 2 due to at least one other criterion	38 (60.3%)	25 (39.7%)	

**Table 5:** Univariate Cox regression based on time to recurrence

Variable	Hazard Ratio	95% Confidence Interval	*P*-value
*Demographics*			
Age	1.02	0.99–1.04	0.17
Female sex	0.8	0.43–1.5	0.49
*Preoperative Tumor Characteristics*			
Preoperative volume	1	0.99–1.01	0.43
Posterior fossa location	0.6	0.08–4.37	0.61
Presence of surrounding edema	0.81	0.25–2.66	0.73
Irregular tumor borders	1.04	0.14–7.6	0.97
Irregular enhancement	0.63	0.09–4.66	0.65
Bone invasion	1.45	0.45–4.73	0.54
*Treatment Data*			
Gross total resection (Simpson Grade 1–2)	0.29	0.14–0.59	**< 0.001**
Residual volume	1.04	1.01–1.07	**0.002**
Adjuvant radiation	0.74	0.26–2.08	0.57
Time to adjuvant radiation, months (mean ± SD)	1.15	0.69–1.91	0.6
*Pathology Data*			
Choroid-type meningioma	1.58	0.56–4.46	0.38
Clear cell-type meningioma	NA	NA	NA
Mitotic count ≥ 4–19 mitoses/10 hpf	1.64	0.88–3.07	0.12
Grade 2 minor criteria	3.31	1.78–6.19	**< 0.001**
Brain invasion	0.58	0.31–1.08	0.087
Deemed Grade 2 due to “brain invasion” alone	0.44	0.2–0.95	**0.036**
Deemed Grade 2 due to “brain invasion” alone, and preoperative volume larger than 25th percentile (16 cc)	0.37	0.16–0.89	**0.027**
Mortality during follow-up period	3.13	1.44–6.83	**0.004**

In multivariable Cox regression (Table 6), GTR remained an independent predictor of lower recurrence risk (HR: 0.29, 95% CI: 0.14–0.59, *p* = 0.001). Grade 2 due to BI alone also maintained its significant association with lower hazard of progression (HR: 0.44, 95% CI: 0.2–0.97, *p* = 0.041).

**Table 6:** Multivariable Cox regression based on time to recurrence

Variable	Hazard Ratio	95% Confidence Interval	*P*-value
Deemed Grade 2 due to “brain invasion” alone	0.44	0.2–0.97	**0.041**
Gross total resection (Simpson Grade 1–2)	0.29	0.14–0.59	**0.001**
Posterior fossa location	1.13	0.15–8.61	0.91

### Sensitivity analyses

Sensitivity analyses further supported our findings (Supplementary Table 1). Reclassifying patients with small tumors (lowest quartile of tumor volume) from the brain invasion-only group to the other Grade 2 criteria group did not change the significance of brain invasion alone as a predictor of reduced hazard of progression (HR: 0.38, 95% CI: 0.16–0.92, *p* = 0.032). Excluding the 33 patients with overlapping Grade 2 criteria or 18 patients that received adjuvant radiation therapy further supports the association of brain invasion alone with a lower hazard of progression. When all sensitivity analyses were performed in combination, BIOB remained a significant predictor of reduced progression risk (HR: 0.29, 95% CI: 0.11–0.78, *p* = 0.014).

Given that adjuvant radiation therapy was administered non-randomly to a minority of patients (18 of 131), its effect on the brain invasion-only group was further evaluated across four model specifications (Supplementary Table 2). The association remained significant whether the radiation therapy term was omitted (HR: 0.44, 95% CI: 0.20–0.96, *p* = 0.040), modeled as a static covariate (HR: 0.43, 95% CI: 0.20–0.95, *p* = 0.037), or modeled as a time-varying covariate to account for the interval between surgery and treatment (HR: 0.44, 95% CI: 0.20–0.97, *p* = 0.042); the association was attenuated but remained consistent in direction when radiation therapy recipients were excluded entirely (HR: 0.47, 95% CI: 0.21–1.05, *p* = 0.067). In a pre-specified multivariable Cox model incorporating age, tumor location, edema, extent of resection, and adjuvant radiation therapy, the association between brain invasion alone and reduced progression risk persisted regardless of whether preoperative tumor volume (HR: 0.44, 95% CI: 0.19–0.98, *p* = 0.046) or residual tumor volume (HR: 0.43, 95% CI: 0.19–0.97, *p* = 0.043) was retained in the model (Supplementary Table 2).

### Illustrative case

We present the case of a 49-year-old woman who presented with progressive visual and olfactory deficits. The patient experienced complete vision loss in the left eye, progressive central vision loss in the right eye, and complete anosmia. She also reported mild word-finding difficulty but denied seizures or severe headaches.

**Fig. 4:**
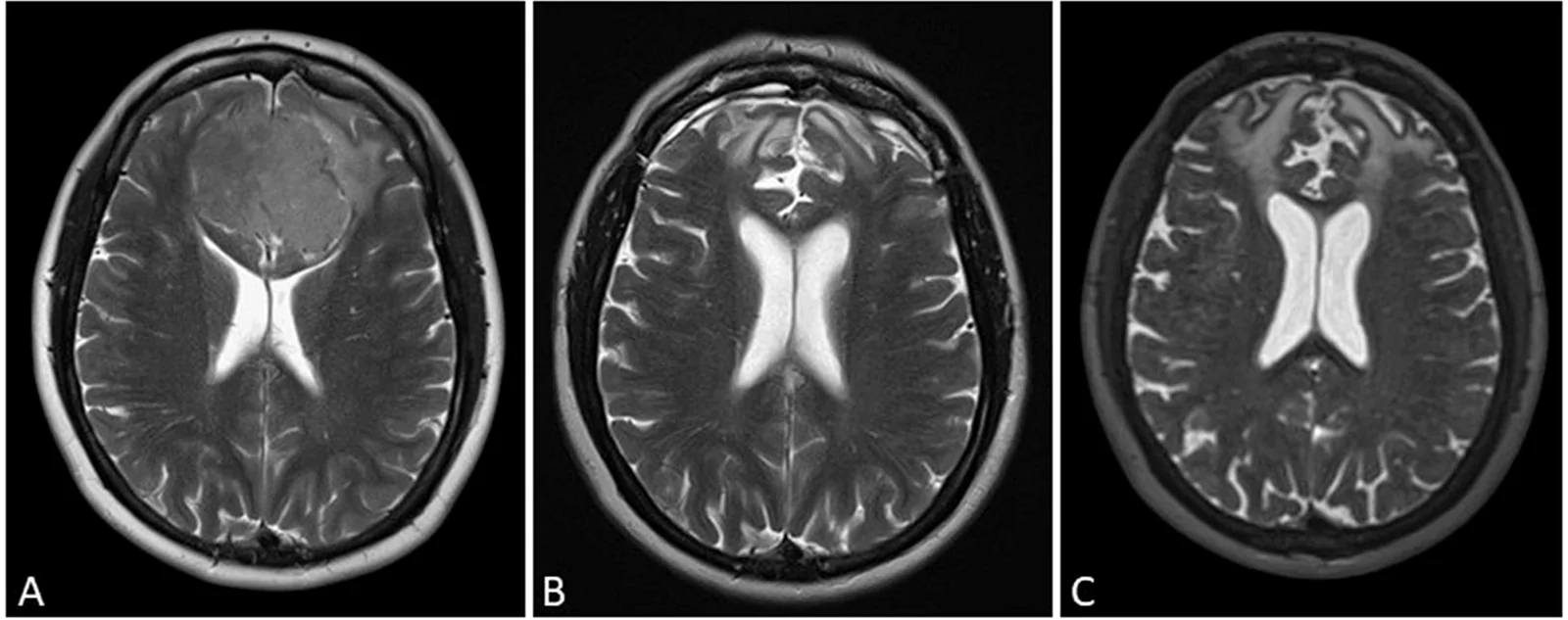
Illustrative case of a > 75th-percentile–volume meningioma classified as Grade 2 due to brain invasion only (BIOB), demonstrating long disease-free survival. A Preoperative axial T2-weighted MRI showing a 5 × 6 × 6 cm planum sphenoidale meningioma extending superiorly in the midline along the cerebral falx. B Postoperative MRI at 5 months showing gross total resection without recurrence. C Follow-up MRI at 107 months (8 years 10 months) demonstrating persistent absence of recurrence

Magnetic resonance imaging revealed a large anterior skull base mass measuring approximately 5 × 6 × 6 cm (estimated volume 87 cc, > 75th percentile), consistent with an olfactory groove meningioma, compressing the optic nerves and chiasm. Preoperative T2-weighted imaging revealed the absence of a defined arachnoid cap suggestive of a breach of the tumor–brain interface (Fig. 4A).

The patient underwent a bifrontal craniotomy and the tumor was carefully dissected from the optic nerves, optic chiasm, and anterior cerebral arteries without vascular injury. Gross total resection was achieved.

Postoperatively, the patient demonstrated marked neurological recovery. Vision improved rapidly, with restoration of functional vision in the right eye and significant overall visual improvement. Notably, she also experienced partial recovery of olfaction, reporting the ability to detect odors such as coffee, indicating improvement from her preoperative complete anosmia. She had no postoperative seizures and recovered without neurological complications.

Postoperative MRI at 5 months confirmed complete tumor resection without evidence of recurrence with the presence of residual encephalomalacia (Fig. 4B). Long-term surveillance imaging at 107 months (8 years and 10 months) demonstrated durable radiographic stability with no recurrence (Fig. 4C).

This case exemplifies how even large meningiomas classified as Grade 2 exclusively on the basis of brain invasion may behave in an indolent manner, consistent with our cohort-level observations that BIOB tumors, especially larger lesions, have significantly lower recurrence risk.

## Discussion

This is a single-center retrospective study that showed that meningiomas with atypical behavior determined by the presence of BIOB have better clinical behavior when compared with tumors that otherwise fulfill Grade 2 criteria. Progression rates were significantly lower in BIOB tumors (17.8% vs. 37.2%), and even TTR was significantly longer after adjustment for known confounders such as extent of resection and tumor location. These data do not support the notion that the presence of BI is an immediate disqualifier for WHO Grade I status.

BI was included as an independent criterion for WHO Grade 2 classification in the 2016 WHO CNS tumor guidelines, on the basis of very limited and retrospective data from a single-center study [6]. The study by Perry et al. (1997), included a cohort of 22 BI-positive meningiomas (mostly collected over 25 years ago) and showed that PFS was significantly shorter in tumors that were BI-positive when compared to the 54 tumors that were BI-negative, despite the absence of any other atypical feature [6]. However, data from several retrospective studies have raised doubts about the prognostic impact of BI, specifically when present in otherwise benign tumors.

The results reported in our study are consistent with those reported in the literature. The study by Spille et al. (2016) included 467 meningiomas, 20 of which were BIOB. The authors found that BIOB meningiomas presented a significantly better prognosis when compared to other WHO Grade II tumors and a prognosis similar to WHO Grade I tumors [10]. Recurrence rates in BI+ (brain invasive) meningiomas were double those observed in non-BI+ (brain non-invasive) meningiomas after GTR but such a difference was not observed for BIOB tumors in particular [10]. The study by Baumgarten et al. (2016) found that BIOB tumors presented a significantly longer PFS when compared to all other WHO Grade II tumors, in agreement with our results [7]. The study by Garcia-Segura et al. (2020) included 181 WHO Grade II meningiomas and also found that BIOB tumors had a significantly better prognosis when compared to all other Grade II tumors. The authors further suggested the combination of BI and necrosis (but not BI alone) as a strong predictor of recurrence [12]. These results support the hypothesis that BI is not clinically significant unless present with other histopathological adverse features.

Although only 14% of patients in our cohort received adjuvant radiation, its administration was non-random and likely enriched for higher-risk tumors; sensitivity analyses accounting for radiation therapy via multiple model specifications (Supplementary Table 2) confirmed that the association between BIOB status and reduced recurrence risk was not attributable to differential radiation exposure. We note, however, that several of these hazard ratios carry wide confidence intervals bordering on non-significance (e.g., HR 0.44, 95% CI 0.20–0.97, for the time-varying radiation therapy model), reflecting the relatively small number of events in our cohort; we therefore advise some caution in interpreting the precision of these estimates, and view the consistency of effect direction across model specifications as more informative than any single point estimate. Regarding the effect of extent of resection, our study showed that GTR was significantly and independently associated with a lower risk of recurrence (HR: 0.29; *p* < 0.001). This result has been shown by a number of studies. Biczok et al. (2019) reported significantly longer PFS after GTR vs. STR (*p* = 0.001), although the difference in PFS between BIOB and WHO Grade I tumors in favor of the latter was not statistically significant [13]. Traylor et al. (2023), in a retrospective analysis of a large cohort of 1,793 meningiomas, which included 90 BIOB meningiomas, also found significantly longer PFS after GTR and no significant difference in recurrence risk between BIOB and WHO Grade I meningiomas [14]. A meta-analysis by Nakasu and Nakasu (2021) pooled data from eight studies to determine the prognostic impact of BI in meningiomas [9]. The authors found that BI was prognostic for recurrence when all tumor grades were analyzed together. However, when data was analyzed for BIOB tumors alone, BI was not a significant predictor of PFS, again supporting the idea that BI does not have prognostic strength in otherwise benign meningiomas. Kim et al. (2022) performed a meta-analysis of 25 studies (3,590 patients) that aimed to determine the association between BI and PFS in meningiomas [15]. The authors found no consistent association between BI and PFS, despite reporting improved PFS after GTR in their own institutional cohort. A study by Banan et al. (2021) did not report a difference in PFS between BIOB tumors and WHO Grade I meningiomas but showed that BIOB tumors had significantly shorter PFS [16]. In this study, the degree of resection was not included as a covariate and was not reported as a significant predictor of recurrence. This omission is notable given that two much larger and more recent cohorts have since suggested why studies like Banan et al.‘s may overstate BI’s independent prognostic weight: Li et al. (2023), in 1,006 meningiomas including 219 BIOB tumors, found brain invasion independently associated with relapse only when co-occurring atypical features and extent of resection were not fully disentangled from BI status itself, positioning BIOB at an intermediate prognosis rather than equivalent to classical Grade 2 disease [17]. More directly, Zhang et al. (2026), in the largest reported single-center cohort to date (1,476 meningiomas, 268 BIOB), found that brain invasion was not an independent predictor of recurrence once mitotic count was included in a combined multivariate model (HR 1.10, *p* = 0.76), while mitotic activity remained strongly significant (HR 3.41, *p* < 0.001); BIOB tumors with two additional atypical features recurred at rates indistinguishable from classical Grade 2 tumors, whereas those with zero or one additional feature behaved like WHO Grade I tumors [18]. Taken together, these data suggest that the adverse prognostic signal attributed to BI in studies such as Banan et al.‘s is more likely carried by unmeasured co-occurring risk factors — extent of resection chief among them. In contrast, a study by Behling et al. (2021) reported a positive correlation between BI and the Ki-67 proliferation index, providing a potential biological link between invasion and proliferation but without direct evidence of clinical behavior in BIOB tumors [19].

The biological plausibility of the notion that BI is a reliable marker of aggressiveness has also been called into question. It has been hypothesized that in larger tumors, BI might occur as a result of the mere physical compression of the brain surface due to the mass effect and mechanical disruption of the pia-glial barrier and not necessarily active infiltrative behavior. This possibility has been discussed in studies by Pizem et al. (2014) and Gousias et al. (2023) and is supported by our findings: in both our size-stratified progression free analysis and our sensitivity analysis, larger tumors (≥ 16 cc), BIOB tumors had a lower progression risk [8, 20]. The concept of BI as a sole reason for a higher-grade classification is complicated by variability in sampling and histological criteria. The histopathologic identification of BI remains a diagnostic challenge, particularly due to its focal nature and the lack of standardized criteria or protocols for sampling. As shown by Dal Col et al., accurate detection of BI often requires extensive sampling, with at least 26 slides necessary to identify BI in 95% of invasive cases [21]. This variability underscores the importance of defining clear morphologic standards and adopting comprehensive sampling strategies to improve grading consistency. Furthermore, studies such as those by Biczok et al. (2019) and Perry (2021) reported high variability in the presence of BI among independent pathologists [6, 13]. The 2021 WHO update on meningioma classification attempted to clarify the criteria required to determine CNS invasion but with potential variable implementation in the field.

### Limitations

This study has several limitations that should be considered when interpreting the results. As a single-center retrospective analysis spanning 20 years, our findings may not be generalizable to other populations or institutional practices. Despite our efforts to standardize pathological assessment through re-grading of all specimens according to the 2016 WHO criteria, the inherent subjectivity in identifying brain invasion remains a challenge in neuropathology, with potential inter-observer variability affecting classification accuracy.

The retrospective design introduces potential selection bias, particularly regarding treatment decisions such as the extent of surgical resection and administration of adjuvant radiation therapy. Although our follow-up period was substantial (mean 74 months), some late progression may have occurred beyond our observation period. Additionally, our sample size, while robust for the primary analysis, becomes more limited in certain subgroup analyses, potentially affecting statistical power. In particular, stratified Cox proportional hazards models within tumor-size subgroups could not be reliably estimated due to limited events and missing data, leading to unstable or non-convergent models.

Changes in surgical techniques, tumor sampling methods, and pathological processing over the two-decade study period may have influenced the consistency of brain invasion detection. Our study also lacks molecular marker data, which are increasingly recognized as important prognostic factors in current meningioma classification schemes. While we controlled for several known confounding variables in our multivariable analysis, unmeasured confounders such as specific molecular subtypes, tumor vascularity, or patient comorbidities could have influenced our results.

Finally, radiographic assessment of progression may have evolved during the study period due to advances in imaging technology and interpretation criteria. These limitations highlight the need for prospective, multi-institutional studies with standardized pathological assessment and molecular profiling to further validate our findings regarding the prognostic significance of brain invasion in meningiomas.

## Conclusion

In conclusion, our study supports the growing body of evidence that BI does not justify an automatic classification to WHO Grade 2. BIOB tumors presented significantly lower progression rates and longer TTR when compared to tumors with other atypical features, and this effect was especially robust among larger tumors (≥ 16 cc), where BIOB tumors showed markedly lower recurrence rates than their OC counterparts. This size-dependent pattern lends support to the hypothesis that BI in larger tumors may, at least in part, reflect mechanical compression of the pia-glial barrier rather than a truly more aggressive infiltrative biology. These findings, along with consistent patterns observed in several retrospective series and multiple meta-analyses, call into question the current grading paradigm. We believe that future revisions of the WHO classification may benefit from a more nuanced and evidence-based approach, which incorporates a combination of histopathological, molecular, and clinical factors, especially tumor size in order to more accurately stratify meningioma risk.

## Supplementary Information

Below is the link to the electronic supplementary material.

Supplementary Material 1

## Data Availability

The data for this study are available upon reasonable request.
