# Supplementary material for: Meningiomas classified as grade 2 due to brain invasion alone have better progression-free survival than other atypical meningiomas
Source: J Neurooncol. 2026 Sep 11;179(2):79. doi: 10.1007/s11060-026-05783-1 (PMC13569586; doi:10.1007/s11060-026-05783-1)
Supplement: Supplementary file 1 [file 11060_2026_5783_MOESM1_ESM.docx]

| **Supplementary Table 1. Sensitivity analyses: transfer of patients with small tumors, exclusion of overlapping criteria, and exclusion of radiation-treated patients. Multivariable Cox regression based on time to recurrence.** | | | |
| --- | --- | --- | --- |
| **Variable** | **Hazard Ratio** | **95% Confidence Interval** | **P-value** |
| *1. Transfer of 11 patients with small tumors (lowest volume quartile) from the BI-only to the OC group.* | | | |
| Deemed Grade 2 due to "brain invasion" alone, and preoperative volume larger than 25th percentile (16 cc) | 0.38 | 0.16 - 0.92 | **0.032** |
| Residual diameter | 1.03 | 1.01 - 1.05 | **0.001** |
| Posterior fossa location | 1.15 | 0.15 - 8.75 | 0.89 |
| *2. Exclusion of 33 OC patients who also had BI.* | | | |
| Deemed Grade 2 due to "brain invasion" alone | 0.38 | 0.16 - 0.87 | **0.022** |
| Gross total resection (Simpson Grade 1-2) | 0.24 | 0.1 - 0.57 | **0.001** |
| Posterior fossa location | 1.22 | 0.16 - 9.4 | 0.851 |
| *3. Exclusion of 18 patients receiving adjuvant radiation.* | | | |
| Deemed Grade 2 due to "brain invasion" alone | 0.47 | 0.21 - 1.05 | 0.067 |
| Gross total resection (Simpson Grade 1-2) | 0.23 | 0.11 - 0.5 | **<0.001** |
| Posterior fossa location | 1.07 | 0.14 - 8.26 | 0.946 |
| *4. All the above.* | | | |
| Deemed Grade 2 due to "brain invasion" alone, and preoperative volume larger than 25th percentile (16 cc) | 0.29 | 0.11 - 0.78 | **0.014** |
| Residual diameter | 0.17 | 0.07 - 0.43 | **<0.001** |
| Posterior fossa location | 1.44 | 0.18 - 11.54 | 0.73 |

| **Supplementary Table 2. Sensitivity analyses: Multivariable Cox regression model comparisons: adjuvant radiation therapy modeling and pre-specified covariate selection. Hazard ratios shown are for the BIOB group.** | | | |
| --- | --- | --- | --- |
| **Model** | **Hazard Ratio** | **95% Confidence Interval** | **P-value** |
| *A. Adjuvant radiation therapy: comparison of model specifications.* | | | |
| A1. No radiation therapy term | 0.44 | 0.20 - 0.96 | **0.040** |
| A2. Radiation therapy as static covariate | 0.43 | 0.20 - 0.95 | **0.037** |
| A3. Exclude radiation therapy patients | 0.47 | 0.21 - 1.05 | 0.067 |
| A4. Radiation therapy as time-varying covariate | 0.44 | 0.20 - 0.97 | **0.042** |
| *A fully stratified model was not pursued given that only 18 of 131 patients received adjuvant radiation therapy.* | | | |
| *B. Pre-specified multivariable Cox model. Preoperative and residual tumor volume were tested in separate models given collinearity with GTR. Both models include age, skull base location, posterior fossa location, edema, GTR, and adjuvant radiation (8 covariates each);* | | | |
| B1. Model retaining preoperative volume | 0.44 | 0.19 - 0.98 | **0.046** |
| B2. Model retaining residual volume | 0.43 | 0.19 - 0.97 | **0.043** |
